# Toxicological impact of microplastics and nanoplastics on humans: understanding the mechanistic aspect of the interaction

**DOI:** 10.3389/ftox.2023.1193386

**Published:** 2023-07-14

**Authors:** Saeed Alqahtani, Shaherah Alqahtani, Quaiser Saquib, Fayaz Mohiddin

**Affiliations:** ^1^ Advanced Diagnostics and Therapeutics Institute, Health Sector, King Abdulaziz City for Science and Technology (KACST), Riyadh, Saudi Arabia; ^2^ Comparative Pathobiology Department, Purdue University Center for Cancer Research, Purdue University, West Lafayette, IN, United States; ^3^ School of Health Sciences, College of Health and Human Sciences, Purdue University, West Lafayette, IN, United States; ^4^ Chair for DNA Research, Zoology Department, College of Science, King Saud University, Riyadh, Saudi Arabia; ^5^ Mountain Research Center for Field Crops, Khudwani, Sher-e-Kashmir University of Agricultural Sciences and Technology, Srinagar, India

**Keywords:** nanoplastics, microplastics, nanoplastic toxicology, COVID-19, PPE, mechanistic aspects of nanoplastics, pollution, plastic waste

## Abstract

Plastic is a pervasive material that has become an indispensable part of our daily lives and is used in various commercial products. However, plastic waste has significantly impacted the environment, accumulating in water and land ecosystems and harming all forms of life. When plastic degrades, it breaks down into smaller particles called microplastics (MPs), which can further breakdown into nanoplastics (NPs). Due to their small size and potential toxicity to humans, NPs are of particular concern. During the COVID-19 pandemic, the production of plastic had reached unprecedented levels, including essential medical kits, food bags, and personal protective equipment (PPE), which generate MPs and NPs when burned. MPs and NPs have been detected in various locations, such as air, food, and soil, but our understanding of their potential adverse health effects is limited. This review aims to provide a comprehensive overview of the sources, interactions, ecotoxicity, routes of exposure, toxicity mechanisms, detection methods, and future directions for the safety evaluation of MPs and NPs. This would improve our understanding of the impact of MPs and NPs on our health and environment and identify ways to address this global crisis.

## Introduction

Plastic is a material that has become an integral part of daily human activities, being used on every occasion. On the other hand, plastic waste, found abundantly in both marine and land ecosystems, has had a profound negative impact on all life forms on our planet. This material is prevalent in an extensive range of commercial products, from cosmetics to construction materials. Plastic waste can float on water, which leads to its transportation to distant regions. Consequently, it accumulates in open oceans, along streamlines, and on the sea floor (Jambeck et al., 2015). In the environment, certain types of plastics breakdown over time into smaller particles, known as microplastics (MPs). First reported in 2004, the threat posed by MPs to human health has since garnered significant attention (Zhang et al., 2022). MPs, composed of synthetic polymer particles, are typically less than 5 mm in size (Ragusa et al., 2021). However, the degradation of plastic does not stop at this stage; MPs can further breakdown into even smaller particles called nanoplastics (NPs) (Dawson et al., 2018). These NPs range in size from 1 nm to 1 μm, a classification that slightly differs from engineered nanomaterials (ENMs) (Gigault et al., 2021). The scientific definition of NPs remains uncertain, with their size classification being disputed as either “<100 nm” or “<1,000 nm” in at least one dimension (Gigault et al., 2018). Due to their minuscule size, NPs can potentially exhibit heightened toxicity, underscoring the need for a comprehensive exploration of their bio-effects.

Additionally, NPs are purposefully incorporated into specific products, such as exfoliating beads in facial scrubs (Hernandez et al., 2017) and industrial pellets (Karlsson et al., 2018). Micro- and nano-plastics (MNPLs) constitute a large portion of plastic contaminants and are ubiquitous in our environment (Shim et al., 2018). Their abundance in aquatic ecosystems ranges from 7.25 × 10^−7^ to 10^2^ particles/L in littoral regions of Africa and Europe (Besseling et al., 2019). Globally, in 2019, over 370 million tons of plastic wastes were either directly or indirectly released into the environment. This number is expected to exceed 12 billion tons by 2050, with less than ten percent of it being recycled (Department, 1950; Geyer et al., 2017). The recycling situation is equally concerning at national level, such as in the United States. According to the most recent data from the EPA in 2020, a mere fraction (8.5%) of the 35.68 million tons of plastic waste generated in the country was recycled. Meanwhile, 15.8% of it was incinerated, and a staggering majority (75.7%) was consigned to landfills in the year 2018 (EPA, 2020). Ultimately, plastic degrades into MPs and NPs, and current recycling methods prove inadequate in removing NPs from the environment. Two primary alternatives to plastic are chitosan, a bioactive polymer, and hemp fiber, a biodegradable polymer. Chitosan is one of the most abundant natural polysaccharides and possesses unique properties, such as non-toxicity, high antibacterial activity, ease of chemical synthesis and modification, and, most importantly, exceptional biodegradability (Hosseinnejad and Jafari, 2016). Given these attributes, chitosan is extensively utilized in industrial and biological applications and presents a plausible alternative to plastic. Hemp fiber, used in producing ropes, polystyrene (PS), flexible building materials, and automobile parts, also offers a sustainable substitute for plastic (Sepe et al., 2018). In recent years, there has been a significant surge in the production and use of plastic containers, such as medical kits, food bags, and personal protective equipment (PPE). Specifically, during the COVID-19 pandemic, the global NP load prompted a drastic increase in plastic production—around 700 million tons in 2020 alone (Shams et al., 2021). This includes disposable face masks, gloves, gowns, COVID-19 kits, food container bags, and eye protectors. Incinerating these plastics whether naturally through brush fires or artificially in incineration plants can lead to the formation of MPs and NPs (Li et al., 2022a; Luo et al., 2022). Previous studies have revealed that MPs and NPs are detected in air, food, soil, and numerous other contexts. However, our current understanding regarding the potential adverse health effects of MPs and NPs on humans is still limited (Trainic et al., 2020; Das et al., 2021; Xie et al., 2022; Yao et al., 2022). Therefore, this review aims to highlight the sources of MPs and NPs, their interaction within the food web and ecotoxicity, routes of human exposure, toxicity mechanisms, detection methods, and future directions to advance our evaluation of their safety.

## Sources of MPs and NPs

Plastic waste and secondary derivatives are the two primary sources of MPs and NPs. Plastic waste is composed mainly of materials such as polyvinyl chloride (PVC), polystyrene, polypropylene (PP), and polyethylene (PE) (Rodrigues et al., 2019). MPs and NPs are released into the environment from the breakdown of plastics including laundry wastewater and tire wear (Guan et al., 2020; Sana et al., 2020; Kiran et al., 2022; Reddy and Nair, 2022). Microfibers shed from synthetic textiles (such as nylon, polyester, and acrylic) significantly contribute to MP pollution, frequently appearing in laundry wastewater. A single wash can shed an average of 7,360 fibers per square meter per liter from polyester fleece fabrics (Carney Almroth et al., 2018). The number and mass of MPs detected in the wastewater from the first wash of polyester and cotton textiles range from 2.1 × 10^5^ to 1.3 × 10^7^ and 0.12–0.33% w/w, respectively (Sillanpää and Sainio, 2017). A substantial amount of MPs and NPs is produced from plastic bags during the tea steeping process, and they often enter the environment through domestic drainage systems and sewage treatment plants (Hernandez et al., 2019). Another source of micro- and nano-scale particles is the particles released from high-speed vehicles when their tires rapidly come in contact with the ground (Kreider et al., 2010). Given the potential adverse effects of MPs and NPs and their significant volume generated from tire wear, this source of plastic pollution requires further investigation.

### Microplastics and nanoplastics in the aquatic food web and ecotoxicity

In aquatic ecosystems, apex predators include species such as large sharks, dolphins, and whales. MPs have been discovered within various fish species’ guts, gills, livers, and brains (Ding et al., 2018). The consumption of hazardous substances and microplastics allows their transfer from one trophic level to the next, leading to bioaccumulation within the food chain. Since MPs do not degrade, they persist within the digestive systems of marine organisms across the entire food chain, inflicting negative biological and physical impacts on marine life (Zhang et al., 2019; Al Mamun et al., 2023). Large fish might not exhibit immediate effects upon ingesting chemically contaminated MPs or NPs, but the gradual accumulation of these particles could eventually prove fatal. Due to a lack of standardized and reliable methods for the sampling, detection, and characterization of MPs and NPs, limited studies have explored the fate of these particles in freshwater environments. The level of toxicity that NPs pose to freshwater ecosystems remains uncertain. The few published studies on this topic are predominantly lab-based and may not replicate the same biological toxicity if conducted in natural environments (Zhang et al., 2022). Laboratory studies have found that exposure to polystyrene nanoplastics (PS-NPs) can lead to a range of toxicological effects, including reproductive abnormalities (Li et al., 2020a; Li et al., 2020b), oxidative stress and gastrointestinal dysfunction (Chae and An, 2017), increased mortality (Liu et al., 2020), growth inhibition and disorders, and neurotoxicity (Zhang et al., 2020). MPs and NPs can be directly ingested by zooplankton, planktivorous fish, and piscivorous fish within the aquatic food web, subsequently moving up the food chain until they are ultimately consumed by humans (Ain Bhutto and You, 2022). The primary sources of particulate plastic ingestion by humans are consuming contaminated seafood, sea salts, and water (Selvam et al., 2020). Toxicological studies have shown that plastic particles within the human gastrointestinal system can have adverse biological effects on digestion and can impair immune function (Chang et al., 2020).

### Human exposure to microplastics and nanoplastics: routes and translocation

#### Ingestion/oral, inhalation, and dermal

Humans are chronically exposed to low concentrations of NPs (Prüst et al., 2020), while all three exposure pathways—ingestion, inhalation, and dermal contact—contribute to the overall presence of MPs and NPs in the human body; the risk of exposure is the highest from seafood and environmental sources. These environments can contain pathogenic microorganisms, long-term weathered polymers, leached chemical additives from polymers, residual monomers, and pollution (Camacho et al., 2019). Recent studies on exposure to and the toxicity of MPs and NPs indicate that ingestion is the primary method through which humans consume plastic particles (Lehner et al., 2019). MPs and NPs can enter the human body by consuming drinking water supplied via plastic pipes (Yong et al., 2020; Kiran et al., 2022). Alarmingly, humans could be exposed to billions of MPs and NPs released from a single plastic tea bag steeped in a beverage (Hernandez et al., 2019). NPs have been detected in table salts and seafood, increasing the risk of oral exposure.

Consequently, prioritizing the advancement of NP detection methods in water and food is imperative (EPoCitF, 2016; González-Fernández et al., 2021; Kaur et al., 2022). Additionally, consuming higher organisms that directly absorb NPs, or depend on lower organisms in the ecological pyramid, provides a broader perspective of oral exposure in humans. MPs and NPs can also enter the human body by consuming foodstuffs contaminated during production processes or packaging (Lau and Wong, 2000; Mason et al., 2018; Du et al., 2020).

Inhalation is another route of human exposure to NPs, which has been detected as a novel carrier for particulate matter (PM2.5 and PM10) (Lai et al., 2022). MPs and NPs in ashes and atmospheric fallout indicate potential human exposure via inhalation (Facciolà et al., 2021; Brandts et al., 2022; Jenner et al., 2022). Wear on car tires can also generate MPs and NPs, releasing them into the surrounding street atmosphere and creating another potential source of inhalation exposure (Kole et al., 2017). Injection as a source of potential exposure has been examined by Tomazic‐Jezic et al. (2001), who reported enhanced phagocytosis in the mouse peritoneal cavity following injection (Tomazic‐Jezic et al., 2001). Studies have detected higher levels of MPs and NPs in indoor atmospheres than outdoor environments, suggesting that humans are exposed to a significant number of NPs (Gaston et al., 2020; Ageel et al., 2022; Yao et al., 2022). Dermal exposure also contributes to human exposure to NPs, especially from those NPs smaller than 40 nm, which have been found to bypass dermal barriers. This is particularly relevant for individuals in close contact with NP-contaminated items, such as personal products, or for those swimming in contaminated water (Rahman et al., 2021; Yee et al., 2021).

#### Translocation of microplastics and nanoplastics

**FIGURE 1:**
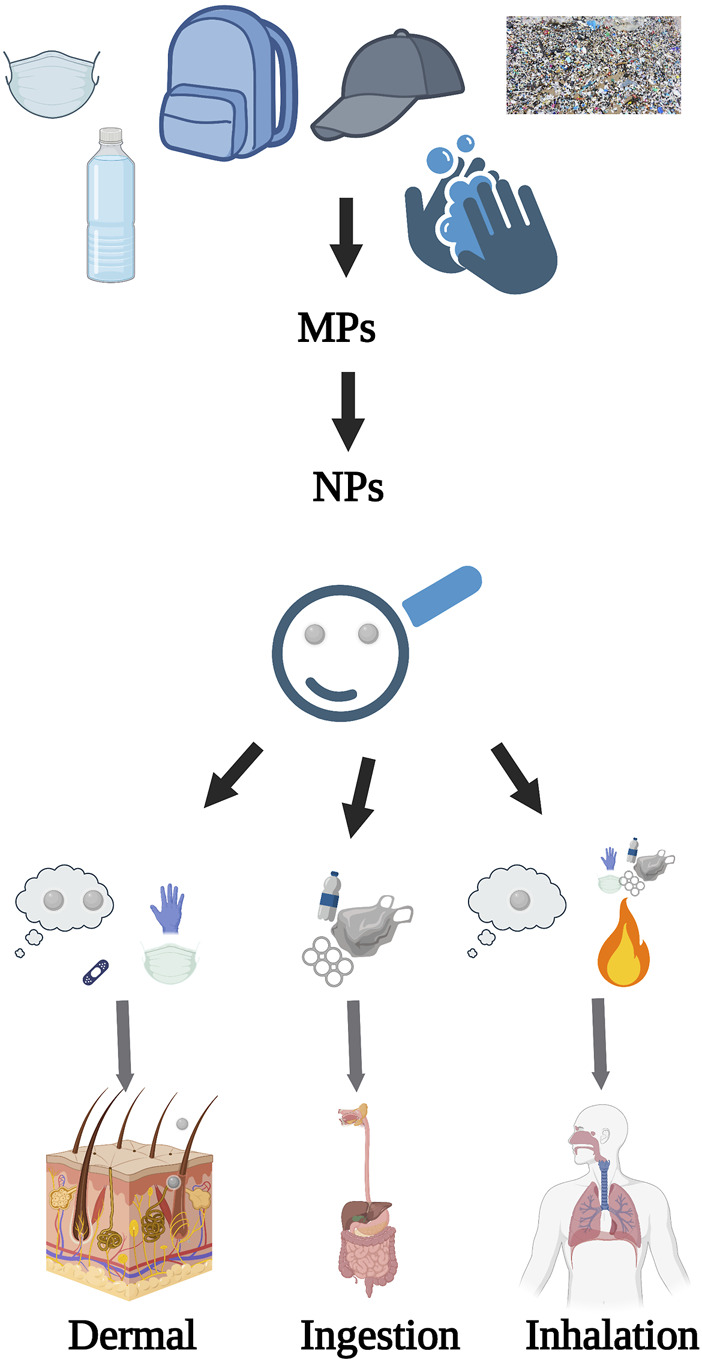
NPs from their formation to translocation. This figure outlines the origin of NPs, their key routes of human exposure, and the potential pathways for their internal movement within the human body, created with BioRender.com.

The translocation and adverse consequences of MPs and NPs on the human body have not been fully investigated. The current understanding is primarily based on laboratory data derived from various test models. A handful of studies have observed the accumulation of MPs and NPs in the intestinal lumen, while others have identified these particles in the fecal matter (Schwabl et al., 2019; Cocca et al., 2020). NPs have been found capable of penetrating and crossing biological barriers, including those of the intestine, lungs, brain, and placenta. Oral exposure to MPs and NPs has shown accumulation in the lumen, with some particles being excreted through the digestive tract (Prüst et al., 2020; Campanale et al., 2020; da Silva Brito et al., 2022). NPs can penetrate the lumen–blood barrier, translocating to blood vessels and distant organs. Some NPs may cross the blood–brain barrier, resulting in brain accumulation. Studies in fish have revealed that NPs can enter the circulatory system, pass the blood–brain barrier, and accumulate in the brain (Guerrera et al., 2021). Additionally, NPs have been observed crossing the placental barrier and have been detected in human placentas (Figure 1) (Ragusa et al., 2021). This capability of NPs raises significant concerns about their potential effects on human health, and in fact, there remains a substantial knowledge gap regarding the health impacts of MPs and NPs on humans (Revel et al., 2018; Catarino et al., 2019). Dong et al. (2023) outlined the mechanism of the translocation of MPs/NPs in animals and connected their translocation to various organotoxic effects based on their exposure through different routes, namely, the dermal, respiratory, and digestive tract (Dong et al., 2023).

#### Mechanism of microplastic and nanoplastic toxicity

Once microplastics and nanoplastics infiltrate a biological milieu, they come into contact and interact with biologically significant macromolecules, such as proteins and lipids. This interaction facilitates the formation of soft and hard NP coronas. The establishment of a corona around NPs triggers a change in their physicochemical properties, thereby influencing their behavior and potentially intensifying the related health implications. Coronas constituted by macromolecules (such as lipids and proteins) regulate the entry and translocation of NPs within cells (Cao et al., 2022). In disease environments, including conditions like metabolic syndrome tied to lipid dysregulation, the constitution of macromolecules differs from that in healthy scenarios. Interestingly, it has been observed that the corona around silver nanoparticles in a metabolic syndrome environment can exacerbate inflammatory responses in mice exhibiting metabolic syndromes compared to their healthy counterparts (Alqahtani et al., 2020; Kobos et al., 2020; Alqahtani et al., 2021; Alqahtani et al., 2022). NPs can be transported and internalized into cells either passively or actively. Passive transport hinges on the potential difference between the concentration of NPs inside and outside the plasma membrane. In contrast, active transport works against the concentration gradient and depends on the ATP consumption. Both these processes occur in tandem to transport and internalize NPs from their surrounding environment (Amobonye et al., 2021; Huang et al., 2021). Under standard physiological conditions, NPs can only passively pass through the cell membrane, provided they fit into the surface pores. An illustrative example can be found in cancer cells, where NPs penetrate and translocate to the cell membrane owing to enlarged surface pores (Bhushan et al., 2017). Similarly, in zebrafish, only smaller NPs can cross the cell membrane through the chorion pore and translocate to other organs (Pitt et al., 2018). Large NPs are mostly blocked by the passive transport mode, which permits only smaller NPs to pass and get transported.

Apart from the aforementioned conditions, several factors (shape, size, corona compounds, surface modification, and cell types) complicate the active mode. Corona compounds such as proteins/lipids are confounders to influencing other factors (Salatin et al., 2015). Within a macrophage, smaller polystyrene particles are internalized via phagocytosis more than the larger ones, leading to the induction of inflammatory gene expression (Olivier et al., 2004). For instance, polyethyleneimine micelles cloaked in a polypeptide corona increased cellular internalization in lymphoblast K562 cells compared to their spherical counterparts. RAW264.7 mice macrophages showed higher internalization of carboxyl-modified nano-polystyrene compared to human endothelial HCMEC cells (dos Santos et al., 2011). The internalization process of NPs into cells often begins with cellular membrane damage and apoptosis. Factors such as the surface charge and types play crucial roles in this process. For instance, polyethylene NPs have been seen to damage the cellular membrane structure, modify its fluidity, and eventually initiate cell death (Rossi et al., 2014). NPs have been observed to breach cellular membranes, triggering intracellular biological effects (Jin et al., 2019; Qu et al., 2019).

The toxicity of NPs is associated with inducing changes in the mitochondria, endoplasmic reticulum, and lysosomes. Prior research has highlighted the harmful effects of NPs on the mitochondrial structure and respiratory function, culminating in metabolic and functional disorders (Armstrong, 2007). An *in vitro* evaluation of human epithelial BEAS-2B cells exposed to NPs revealed significant functional changes, including abnormal energy metabolism (Carney Almroth et al., 2018). NPs have been noted to modify the mitochondrial function by increasing oxygen consumption in zebrafish models (Pitt et al., 2018). Exposure to NPs has been linked to anti-apoptotic signaling of Bcl-2–caspase8 in the *C. elegans* model (Qu et al., 2019). In *Sterechinus neumayeri* cells, NP exposure led to increased levels of antioxidant activity, including catalase, superoxide, metallothionein, and anti-apoptotic signaling of Bcl-2–caspase8 (Bergami et al., 2019). NPs have been associated with the induction of oxidative stress and stress-related autophagy pathways in the endoplasmic reticulum, leading to the upregulation of the Grp78 and Grp170 expression in coelomocytes (Bergami et al., 2019).

**FIGURE 2:**
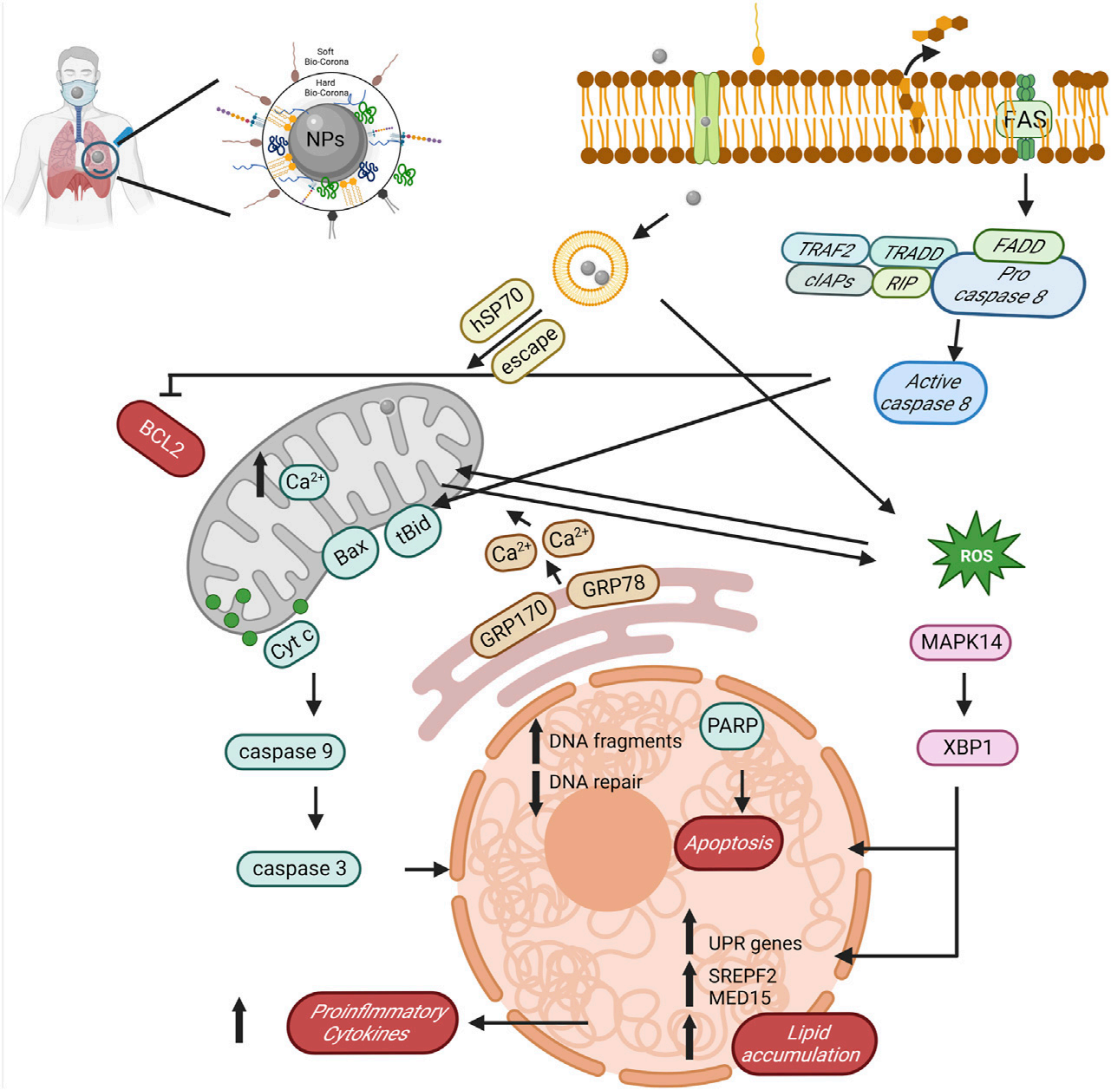
The potential role and mechanism of biocorona formation on NPs in mediating toxicity. The diagram reveals the sequential actions initiated by NPs, such as BCL2 suppression, stimulation of caspase activity (both 8 and 9), induction of reactive oxygen species, amplification of proinflammatory cytokines, lipid accretion, and ultimately, apoptosis, created with BioRender.com.

After exposure to NPs, *C. elegans* exhibited endoplasmic reticulum (ER) stress, unfolded protein responses, and a disrupted fat metabolism. This involved the phosphorylation of MAPK14 and upregulation of XBP1, sparking an innate immune response. NPs appear to regulate an autophagy mechanism through the endoplasmic reticulum stress instigated by misfolded protein aggregation (Qu et al., 2019; Yang et al., 2020). These indicate the important role of the ER in responding to biological effects triggered by NPs. NPs have also been found to internalize and accumulate within lysosomes, leading to lysosomal dysfunction by inducing an acidic pH and modifying the membrane integrity. This accumulation of NPs triggers the transcription factor EB, enhancing the lysosome–autophagosome fusion and the clearance of autophagic cargo (Song et al., 2015; Saftig and Haas, 2016). An unresolved blockage of autophagic flux may ultimately result in cell death by damaging lysosomes (Wang et al., 2018). NPs have been reported of forming coronas, internalizing in the lysosome, and causing damage upon the degradation of their surface corona (Wang et al., 2013). For instance, PS NPs have been found to accumulate in lysosomes and cause membrane damage (Brandts et al., 2020) (Figure 2).

IL-8 expression has been found to increase in response to polystyrene particles with a size range of 202–535 nm, triggering inflammatory effects on human A549 lung cells (Brown et al., 2001). It has been noted that unaltered/carboxylated PS NPs with particle sizes of 20 nm, 44 nm, 500 nm, and 1,000 nm increase the expression of IL-6 and IL-8 and inflammation in various types of human cancers (Prietl et al., 2014; Forte et al., 2016). Carboxylated and amino-modified PS particles are noted to alter the scavenger receptor expression in human cells, boost IL-10 production in M2 cells, and elevate TGF-1 (M1) and energy metabolism (M2) (Fuchs et al., 2016). Unaltered polythene particles with particle sizes of 0.3 µm and 10 µm increased the secretion of IL-6, IL-1B, and TNF in murine macrophages (Green et al., 1998). Polyethylene particles from plastic prosthetic implants cause liver inflammation and periprosthetic bone resorption (Devane et al., 1995a; Devane et al., 1995b; Nich and Goodman, 2014). Additionally, 5 μm and 20 µm PS-MPs adversely affect neurotransmission (Deng et al., 2017).

Children are more likely to develop metabolic disorders due to exposure to MPs of sizes 0.5 and 5 μm, contributing to gut microbiota dysbiosis and barrier dysfunction (Luo et al., 2019a; Luo et al., 2019b). Pristine and fluorescent PS-MPs between 5 and 20 µm in size alter the amino acid and bile acid metabolism, impair energy metabolism, and cause dysbiosis of the gut microbiota and dysfunction of the intestinal barrier (Deng et al., 2017; Lu et al., 2018; Luo et al., 2019a; Jin et al., 2019; Stock et al., 2019). PS NPs (30 nm) prevented the movement of vesicles and the distribution of proteins related to cytokinesis (Xia et al., 2020). Basolateral K^+^ ion channels are activated by anionic carboxylated PS NPs of 20 nm size, which also cause the Cl- and HCO_3_ ion efflux (McCarthy et al., 2011). The sizes of 50 nm and 200 nm cationic PS NPs interfere with the intestinal ion transport and cellular uptake (Mahler et al., 2012).

#### Detection of microplastics and nanoplastics

With the tremendous number of NPs released into the environment, investigating their safety on human health is necessary. These evaluations first focus on detecting MPs and NPs in the environment. The standard available method to detect NPs is asymmetric flow field-flow fractionation coupled with angle light scattering (MALS), fluorescent labeling, and Raman tweezers (Correia and Loeschner, 2018; Catarino et al., 2019; Gillibert et al., 2019). However, due to issues with fluorescent labeling and cellular autofluorescence leakage, artifacts often appear among the particles, leading to false positive results in biological samples. There is a pressing need to develop a comprehensive suite of detection techniques that are efficient, convenient, and accurate for effective environmental management and safeguarding human health from exposure to these NPs. Achieving this goal would empower toxicologists to accurately detect NPs in biological systems, facilitate the establishment of clear standards, and enable the proactive notification of those at high risk of exposure. For instance, epidemiological and murine studies have suggested that individuals with pre-existing conditions, such as metabolic syndromes, are more susceptible to engineered nanoparticles’ impact than healthier individuals (Kobos et al., 2019; Alqahtani et al., 2020; Kobos et al., 2020; Alqahtani et al., 2021; Alqahtani et al., 2022; Xia et al., 2022). Similar vulnerabilities may well extend to the exposure to NPs. Even though removing NPs released into the environment poses significant challenges, potential methods to reduce the volume of these discharged NPs do exist and can be explored further.

### Future directions in advancing microplastic and nanoplastic safety evaluation

The mitigation of environmental pollutants, including NPs, typically employs physical, chemical, or biological means. Although physical and chemical methods have been explored, they tend to produce more NPs rather than reducing the existing ones. Biodegradation emerges as the sole effective strategy for eradicating NP pollution. A substantial challenge confronting toxicologists and policymakers is understanding the lifespan of NPs once introduced into the environment. Since NPs can absorb and leach environmental chemicals during transit, they can transform under varied conditions and media (Jeong et al., 2023).

Furthermore, aged NPs may change their chemical and physical properties, possibly causing the release of additives into the surroundings. Despite an unclear comprehensive impact, this could potentially lead to adverse consequences for humans and the environment, necessitating an investigation into the effects of the chemicals released from NPs. Another challenge is establishing rational and relevant NP concentrations for testing, as many toxicity evaluations have been conducted with non-representative exposure levels. Hence, assessments should consider the potential harm of NPs at environmentally realistic concentrations. Significantly, vast quantities of NPs have been detected in the atmosphere, while studies investigating the potential respiratory effects remain sparse.

In conclusion, standardizing NP characterization is crucial to guarantee reproducible and compatible toxicological evaluations. Bodies like the Organization for Economic Cooperation and Development (OECD) and the International Organization for Standardization (ISO) are in the process of developing standards for aspects including NP synthesis methods, size determination, surface properties, and analytical techniques (OECD, 2017). Developing suitable *in vitro* and *in vivo* models is essential for assessing potential NP toxicity, incorporating marine and zebrafish models to probe the systemic impacts of NPs and *in vitro* models in investigating their toxicological mechanisms (Mattsson et al., 2017). Given that animal models cannot entirely represent human characteristics and responses to microplastics and NPs, the application of human organoids may offer a more relevant toxicity assessment model in future perspectives (Li et al., 2022b; Bredeck et al., 2022; Chandy et al., 2022; Cheng et al., 2022; Li et al., 2022c; Hou et al., 2022). A better understanding of NPs’ behavior would aid in identifying potential exposure pathways and shaping assessment strategies. Attributes such as the size, surface characteristics, and aggregation state of NPs can influence their interaction with environmental elements, such as soil, water, and biota (Cui et al., 2021). Assessing the potential toxicity of NPs on the microbiome, which is pivotal for human health and ecosystem functioning, is also necessary (Li et al., 2021). To minimize potential risks associated with NPs, a comprehensive risk management strategy should be devised, which could encompass reducing nanoplastic emissions, developing alternative materials, and improving waste management practices (Oberoi and Garg, 2021). Gaining insights into the mechanisms of MPs and NPs in animals could enhance efforts toward plastic elimination and potentially reduce organotoxicity associated with MPs and NPs, moving toward a life free of plastic pollution.

## Conclusion

While there are extensive studies on MPs and NPs in marine environments, our understanding of their human exposure pathways is still limited. Based on existing evidence, it is apparent that humans can be exposed to MPs and NPs via ingestion, inhalation, and dermal routes. Following exposure, MPs and NPs can cross biological barriers, potentially inducing toxicity that triggers oxidative stress, inflammatory reactions, and metabolism disorders, particularly gastrointestinal and pulmonary infections. Although this review provides insight into the potential mechanisms of MP and NP toxicity in humans, more research is needed on the bioaccumulation, distribution, and transcriptomic changes caused by MP and NP inappropriate test models. Moreover, human biomonitoring studies are crucial in determining MPs and NPs’ presence in biological fluids. This would offer a comprehensive understanding and possibly unravel any associated health issues.

## References

[B1] Ageel H. K. Harrad S. Abdallah M. A-E. (2022). Occurrence, human exposure, and risk of microplastics in the indoor environment. Environ. Sci. Process. Impacts 24 (1), 17–31. 10.1039/d1em00301a 34842877

[B2] Ain Bhutto S. U. You X. (2022). Spatial distribution of microplastics in Chinese freshwater ecosystem and impacts on food webs. Environ. Pollut. 293, 118494. 10.1016/j.envpol.2021.118494 34780753

[B3] Al Mamun A. Prasetya T. A. E. Dewi I. R. Ahmad M. (2023). Microplastics in human food chains: Food becoming a threat to health safety. Sci. Total Environ. 858, 159834. 10.1016/j.scitotenv.2022.159834 36461575

[B4] Alqahtani S. Kobos L. M. Xia L. Ferreira C. Franco J. Du X. (2020). Exacerbation of nanoparticle-induced acute pulmonary inflammation in a mouse model of metabolic syndrome. Front. Immunol. 11, 818. 10.3389/fimmu.2020.00818 32457752 PMC7221136

[B5] Alqahtani S. Xia L. Jannasch A. Ferreira C. Franco J. Shannahan J. H. (2021). Disruption of pulmonary resolution mediators contribute to exacerbated silver nanoparticle-induced acute inflammation in a metabolic syndrome mouse model. Toxicol. Appl. Pharmacol. 431, 115730. 10.1016/j.taap.2021.115730 34601004 PMC8545917

[B6] Alqahtani S. Xia L. Shannahan J. H. (2022). Enhanced silver nanoparticle-induced pulmonary inflammation in a metabolic syndrome mouse model and resolvin D1 treatment. Part Fibre Toxicol. 19 (1), 54. 10.1186/s12989-022-00495-6 35933425 PMC9356467

[B7] Amobonye A. Bhagwat P. Singh S. Pillai S. (2021). Plastic biodegradation: Frontline microbes and their enzymes. Sci. Total Environ. 759, 143536. 10.1016/j.scitotenv.2020.143536 33190901

[B8] Armstrong J. S. (2007). Mitochondrial medicine: Pharmacological targeting of mitochondria in disease. Br. J. Pharmacol. 151 (8), 1154–1165. 10.1038/sj.bjp.0707288 17519949 PMC2189819

[B9] Bergami E. Krupinski Emerenciano A. González-Aravena M. Cárdenas C. Hernández P. Silva J. (2019). Polystyrene nanoparticles affect the innate immune system of the Antarctic sea urchin *Sterechinus neumayeri* . Polar Biol. 42, 743–757. 10.1007/s00300-019-02468-6

[B10] Besseling E. Redondo-Hasselerharm P. Foekema E. M. Koelmans A. A. (2019). Quantifying ecological risks of aquatic micro- and nanoplastic. Crit. Rev. Environ. Sci. Technol. 49 (1), 32–80. 10.1080/10643389.2018.1531688

[B11] Bhushan B. Khanadeev V. Khlebtsov B. Khlebtsov N. Gopinath P. (2017). Impact of albumin based approaches in nanomedicine: Imaging, targeting and drug delivery. Adv. Colloid Interface Sci. 246, 13–39. 10.1016/j.cis.2017.06.012 28716187

[B12] Brandts I. Cánovas M. Tvarijonaviciute A. Llorca M. Vega A. Farré M. (2022). Nanoplastics are bioaccumulated in fish liver and muscle and cause DNA damage after a chronic exposure. Environ. Res. 212, 113433. 10.1016/j.envres.2022.113433 35580665

[B13] Brandts I. Garcia-Ordoñez M. Tort L. Teles M. Roher N. (2020). Polystyrene nanoplastics accumulate in ZFL cell lysosomes and in zebrafish larvae after acute exposure, inducing a synergistic immune response *in vitro* without affecting larval survival *in vivo* . Environ. Sci. Nano 7 (8), 2410–2422. 10.1039/d0en00553c

[B14] Bredeck G. Halamoda-Kenzaoui B. Bogni A. Lipsa D. Bremer-Hoffmann S. (2022). Tiered testing of micro- and nanoplastics using intestinal *in vitro* models to support hazard assessments. Environ. Int. 158, 106921. 10.1016/j.envint.2021.106921 34634620

[B15] Brown D. M. Wilson M. R. MacNee W. Stone V. Donaldson K. (2001). Size-Dependent proinflammatory effects of ultrafine polystyrene particles: A role for surface area and oxidative stress in the enhanced activity of ultrafines. Toxicol. Appl. Pharmacol. 175 (3), 191–199. 10.1006/taap.2001.9240 11559017

[B16] Camacho M. Herrera A. Gómez M. Acosta-Dacal A. Martínez I. Henríquez-Hernández L. A. (2019). Organic pollutants in marine plastic debris from Canary Islands beaches. Sci. Total Environ. 662, 22–31. 10.1016/j.scitotenv.2018.12.422 30684899

[B17] Campanale C. Massarelli C. Savino I. Locaputo V. Uricchio V. F. (2020). A detailed review study on potential effects of microplastics and additives of concern on human health. Int. J. Environ. Res. Public Health 17 (4), 1212. 10.3390/ijerph17041212 32069998 PMC7068600

[B18] Cao J. Yang Q. Jiang J. Dalu T. Kadushkin A. Singh J. (2022). Coronas of micro/nano plastics: A key determinant in their risk assessments. Part Fibre Toxicol. 19 (1), 55. 10.1186/s12989-022-00492-9 35933442 PMC9356472

[B19] Carney Almroth B. M. Åström L. Roslund S. Petersson H. Johansson M. Persson N-K. (2018). Quantifying shedding of synthetic fibers from textiles; a source of microplastics released into the environment. Environ. Sci. Pollut. Res. 25 (2), 1191–1199. 10.1007/s11356-017-0528-7 PMC5766707 29081044

[B20] Catarino A. I. Frutos A. Henry T. B. (2019). Use of fluorescent-labelled nanoplastics (NPs) to demonstrate NP absorption is inconclusive without adequate controls. Sci. Total Environ. 670, 915–920. 10.1016/j.scitotenv.2019.03.194 30921723

[B21] Chae Y. An Y-J. (2017). Effects of micro- and nanoplastics on aquatic ecosystems: Current research trends and perspectives. Mar. Pollut. Bull. 124 (2), 624–632. 10.1016/j.marpolbul.2017.01.070 28222864

[B22] Chandy M. Obal D. Wu J. C. (2022). Elucidating effects of environmental exposure using human-induced pluripotent stem cell disease modeling. EMBO Mol. Med. 14 (11), e13260. 10.15252/emmm.202013260 36285490 PMC9641419

[B23] Chang X. Xue Y. Li J. Zou L. Tang M. (2020). Potential health impact of environmental micro- and nanoplastics pollution. J. Appl. Toxicol. 40 (1), 4–15. 10.1002/jat.3915 31828819

[B24] Cheng W. Li X. Zhou Y. Yu H. Xie Y. Guo H. (2022). Polystyrene microplastics induce hepatotoxicity and disrupt lipid metabolism in the liver organoids. Sci. Total Environ. 806 (Pt 1), 150328. 10.1016/j.scitotenv.2021.150328 34571217

[B25] Cocca M. Di Pace E. Errico M. E. Gentile G. Montarsolo A. Mossotti R. (2020). Proceedings of the 2nd international conference on microplastic pollution in the mediterranean sea. Springer Nature.

[B26] Correia M. Loeschner K. (2018). Detection of nanoplastics in food by asymmetric flow field-flow fractionation coupled to multi-angle light scattering: Possibilities, challenges and analytical limitations. Anal. Bioanal. Chem. 410 (22), 5603–5615. 10.1007/s00216-018-0919-8 29411085

[B27] Cui L. Wang X. Li J. Gao X. Zhang J. Liu Z. (2021). Ecological and health risk assessments and water quality criteria of heavy metals in the Haihe River. Environ. Pollut. 290, 117971. 10.1016/j.envpol.2021.117971 34438170

[B28] da Silva Brito W. A. Mutter F. Wende K. Cecchini A. L. Schmidt A. Bekeschus S. (2022). Consequences of nano and microplastic exposure in rodent models: The known and unknown. Part Fibre Toxicol. 19 (1), 28. 10.1186/s12989-022-00473-y 35449034 PMC9027452

[B29] Das G. Das T. Chowdhury N. Chatterjee D. Bagchi A. Ghosh Z. (2021). Repurposed drugs and nutraceuticals targeting envelope protein: A possible therapeutic strategy against COVID-19. Genomics 113(1), 1129–1140. 10.1016/j.ygeno.2020.11.009 33189776 PMC7661923

[B30] Dawson A. L. Kawaguchi S. King C. K. Townsend K. A. King R. Huston W. M. (2018). Turning microplastics into nanoplastics through digestive fragmentation by Antarctic krill. Nat. Commun. 9 (1), 1001. 10.1038/s41467-018-03465-9 29520086 PMC5843626

[B31] Deng Y. Zhang Y. Lemos B. Ren H. (2017) Tissue accumulation of microplastics in mice and biomarker responses suggest widespread health risks of exposure. Sci. Rep. 7, 46687. 10.1038/srep46687 28436478 PMC5402289

[B32] Department S. R. Annual production of plastics worldwide from 1950 to 2021. *Stat.* Mar. 24, 2023.

[B33] Devane P. A. Bourne R. B. Rorabeck C. H. Hardie R. M. Horne J. G. (1995a). Measurement of polyethylene wear in metal-backed acetabular cups. I. Three-dimensional technique. Clin. Orthop. Relat. Res. 319, 303–316. 10.1097/00003086-199510000-00033 7554644

[B34] Devane P. A. Bourne R. B. Rorabeck C. H. MacDonald S. Robinson E. J. (1995b). Measurement of polyethylene wear in metal-backed acetabular cups. II. Clinical application. Clin. Orthop. Relat. Res. 319, 317–326. 10.1097/00003086-199510000-00034 7554645

[B35] Ding J. Zhang S. Razanajatovo R. M. Zou H. Zhu W. (2018). Accumulation, tissue distribution, and biochemical effects of polystyrene microplastics in the freshwater fish red tilapia (*Oreochromis niloticus*). Environ. Pollut. 238, 1–9. 10.1016/j.envpol.2018.03.001 29529477

[B36] Dong X. Liu X. Hou Q. Wang Z. (2023). From natural environment to animal tissues: A review of microplastics (nanoplastics) translocation and hazards studies. Sci. Total Environ. 855, 158686. 10.1016/j.scitotenv.2022.158686 36099943

[B37] dos Santos T. Varela J. Lynch I. Salvati A. Dawson K. A. (2011). Quantitative assessment of the comparative nanoparticle-uptake efficiency of a range of cell lines. Small 7 (23), 3341–3349. 10.1002/smll.201101076 22009913

[B38] Du F. Cai H. Zhang Q. Chen Q. Shi H. (2020). Microplastics in take-out food containers. J. Hazard Mater 399, 122969. 10.1016/j.jhazmat.2020.122969 32526446

[B39] EPA (2020). Advancing sustainable materials management:2018 fact sheet december 2020.

[B40] EpoCitF C. (2016). Presence of microplastics and nanoplastics in food, with particular focus on seafood. Efsa J. 14 (6), e04501. 10.2903/j.efsa.2016.4501 PMC11847996 40007823

[B41] Facciolà A. Visalli G. Pruiti Ciarello M. Di Pietro A. (2021). Newly emerging airborne pollutants: Current knowledge of health impact of micro and nanoplastics. Int. J. Environ. Res. Public Health 18 (6), 2997. 10.3390/ijerph18062997 33803962 PMC7998604

[B42] Forte M. Iachetta G. Tussellino M. Carotenuto R. Prisco M. De Falco M. (2016). Polystyrene nanoparticles internalization in human gastric adenocarcinoma cells. Toxicol Vitro 31, 126–136. 10.1016/j.tiv.2015.11.006 26585375

[B43] Fuchs A-K. Syrovets T. Haas K. A. Loos C. Musyanovych A. Mailänder V. (2016). Carboxyl- and amino-functionalized polystyrene nanoparticles differentially affect the polarization profile of M1 and M2 macrophage subsets. Biomaterials 85, 78–87. 10.1016/j.biomaterials.2016.01.064 26854393

[B44] Gaston E. Woo M. Steele C. Sukumaran S. Anderson S. (2020). Microplastics differ between indoor and outdoor air masses: Insights from multiple microscopy methodologies. Appl. Spectrosc. 74 (9), 1079–1098. 10.1177/0003702820920652 32233850

[B45] Geyer R. Jambeck J. R. Law K. L. (2017). Production, use, and fate of all plastics ever made. Sci. Adv. 3 (7), e1700782. 10.1126/sciadv.1700782 28776036 PMC5517107

[B46] Gigault J. El Hadri H. Nguyen B. Grassl B. Rowenczyk L. Tufenkji N. (2021). Nanoplastics are neither microplastics nor engineered nanoparticles. Nat. Nanotechnol. 16 (5), 501–507. 10.1038/s41565-021-00886-4 33927364

[B47] Gigault J. Halle At Baudrimont M. Pascal P-Y. Gauffre F. Phi T-L. (2018) Current opinion: What is a nanoplastic? Environ. Pollut. 235, 1030–1034. 10.1016/j.envpol.2018.01.024 29370948

[B48] Gillibert R. Balakrishnan G. Deshoules Q. Tardivel M. Magazzù A. Donato M. G. (2019). Raman tweezers for small microplastics and nanoplastics identification in seawater. Environ. Sci. Technol. 53 (15), 9003–9013. 10.1021/acs.est.9b03105 31259538

[B49] González-Fernández C. Díaz Baños F. G. Esteban M. Á. Cuesta A. (2021). Functionalized nanoplastics (NPs) increase the toxicity of metals in fish cell lines. Int. J. Mol. Sci. 22, 7141. 10.3390/ijms22137141 34281191 PMC8268098

[B50] Green T. R. Fisher J. Stone M. Wroblewski B. M. Ingham E. (1998). Polyethylene particles of a ‘critical size’ are necessary for the induction of cytokines by macrophages *in vitro* . Biomaterials 19 (24), 2297–2302. 10.1016/s0142-9612(98)00140-9 9884043

[B51] Guan W-j. Ni Z-y. Hu Y. Liang W-h. Ou C-q. He J-x. (2020). Clinical characteristics of coronavirus disease 2019 in China. N. Engl. J. Med. 382 (18), 1708–1720. 10.1056/NEJMoa2002032 32109013 PMC7092819

[B52] Guerrera M. C. Aragona M. Porcino C. Fazio F. Laurà R. Levanti M. (2021). Micro and nano plastics distribution in fish as model organisms: Histopathology, blood response and bioaccumulation in different organs. Appl. Sci. 11, 5768. 10.3390/app11135768

[B53] Hernandez L. M. Xu E. G. Larsson H. C. E. Tahara R. Maisuria V. B. Tufenkji N. (2019). Plastic teabags release billions of microparticles and nanoparticles into tea. Environ. Sci. Technol. 53 (21), 12300–12310. 10.1021/acs.est.9b02540 31552738

[B54] Hernandez L. M. Yousefi N. Tufenkji N. (2017). Are there nanoplastics in your personal care products? Environ. Sci. Technol. Lett. 4 (7), 280–285. 10.1021/acs.estlett.7b00187

[B55] Hosseinnejad M. Jafari S. M. (2016). Evaluation of different factors affecting antimicrobial properties of chitosan. Int. J. Biol. Macromol. 85, 467–475. 10.1016/j.ijbiomac.2016.01.022 26780706

[B56] Hou Z. Meng R. Chen G. Lai T. Qing R. Hao S. (2022). Distinct accumulation of nanoplastics in human intestinal organoids. Sci. Total Environ. 838 (Pt 2), 155811. 10.1016/j.scitotenv.2022.155811 35597345

[B57] Huang D. Tao J. Cheng M. Deng R. Chen S. Yin L. (2021). Microplastics and nanoplastics in the environment: Macroscopic transport and effects on creatures. J. Hazard. Mater. 407, 124399. 10.1016/j.jhazmat.2020.124399 33191019

[B58] Jambeck J. R. Geyer R. Wilcox C. Siegler T. R. Perryman M. Andrady A. (2015). Marine pollution. Plastic waste inputs from land into the ocean. Science 347 (6223), 768–771. 10.1126/science.1260352 25678662

[B59] Jenner L. C. Rotchell J. M. Bennett R. T. Cowen M. Tentzeris V. Sadofsky L. R. (2022). Detection of microplastics in human lung tissue using μFTIR spectroscopy. Sci. Total Environ. 831, 154907. 10.1016/j.scitotenv.2022.154907 35364151

[B60] Jeong Y. Gong G. Lee H-J. Seong J. Hong S. W. Lee C. (2023). Transformation of microplastics by oxidative water and wastewater treatment processes: A critical review. J. Hazard. Mater. 443, 130313. 10.1016/j.jhazmat.2022.130313 36372022

[B61] Jin Y. Lu L. Tu W. Luo T. Fu Z. (2019). Impacts of polystyrene microplastic on the gut barrier, microbiota and metabolism of mice. Sci. Total Environ. 649, 308–317. 10.1016/j.scitotenv.2018.08.353 30176444

[B62] Karlsson T. M. Arneborg L. Broström G. Almroth B. C. Gipperth L. Hassellöv M. (2018). The unaccountability case of plastic pellet pollution. Mar. Pollut. Bull. 129 (1), 52–60. 10.1016/j.marpolbul.2018.01.041 29680567

[B63] Kaur H. Rawat D. Poria P. Sharma U. Gibert Y. Ethayathulla A. S. (2022). Ecotoxic effects of microplastics and contaminated microplastics – emerging evidence and perspective. Sci. Total Environ. 841, 156593. 10.1016/j.scitotenv.2022.156593 35690218

[B64] Kiran B. R. Kopperi H. Venkata Mohan S. (2022). Micro/nano-plastics occurrence, identification, risk analysis and mitigation: Challenges and perspectives. Rev. Environ. Sci. Bio/Technology 21 (1), 169–203. 10.1007/s11157-021-09609-6 PMC8792138 35103051

[B65] Kobos L. Alqahtani S. Xia L. Coltellino V. Kishman R. McIlrath D. (2020). Comparison of silver nanoparticle-induced inflammatory responses between healthy and metabolic syndrome mouse models. J. Toxicol. Environ. Health, Part A 83 (7), 249–268. 10.1080/15287394.2020.1748779 PMC7493428 32281499

[B66] Kobos L. M. Alqahtani S. Ferreira C. R. Aryal U. K. Hedrick V. Sobreira T. J. P. (2019). An integrative proteomic/lipidomic analysis of the gold nanoparticle Biocorona in healthy and obese conditions. Appl. Vitro Toxicol. 5 (3), 150–166. 10.1089/aivt.2019.0005 PMC6768227 32292798

[B67] Kole P. J. Löhr A. J. Van Belleghem F. Ragas A. M. J. (2017). Wear and tear of tyres: A stealthy source of microplastics in the environment. Int. J. Environ. Res. Public Health 14 (10), 1265. 10.3390/ijerph14101265 29053641 PMC5664766

[B68] Kreider M. L. Panko J. M. McAtee B. L. Sweet L. I. Finley B. L. (2010). Physical and chemical characterization of tire-related particles: Comparison of particles generated using different methodologies. Sci. Total Environ. 408 (3), 652–659. 10.1016/j.scitotenv.2009.10.016 19896165

[B69] Lai H. Liu X. Qu M. (2022). Nanoplastics and human health: Hazard identification and biointerface. Nanomater. (Basel) 12 (8), 1298. 10.3390/nano12081298 PMC9026096 35458006

[B70] Lau O-W. Wong S-K. (2000). Contamination in food from packaging material. J. Chromatogr. A 882 (1), 255–270. 10.1016/s0021-9673(00)00356-3 10895950

[B71] Lehner R. Weder C. Petri-Fink A. Rothen-Rutishauser B. (2019). Emergence of nanoplastic in the environment and possible impact on human health. Environ. Sci. Technol. 53 (4), 1748–1765. 10.1021/acs.est.8b05512 30629421

[B72] Li D. Deng Y. Wang S. Du H. Xiao G. Wang D. (2020a). Assessment of nanopolystyrene toxicity under fungal infection condition in *Caenorhabditis elegans* . Ecotoxicol. Environ. Saf. 197, 110625. 10.1016/j.ecoenv.2020.110625 32302863

[B73] Li D. Ji J. Yuan Y. Wang D. (2020b). Toxicity comparison of nanopolystyrene with three metal oxide nanoparticles in nematode *Caenorhabditis elegans* . Chemosphere 245, 125625. 10.1016/j.chemosphere.2019.125625 31855754

[B74] Li H. Wu Z-F. Yang X-R. An X-L. Ren Y. Su J-Q. (2021). Urban greenness and plant species are key factors in shaping air microbiomes and reducing airborne pathogens. Environ. Int. 153, 106539. 10.1016/j.envint.2021.106539 33813232

[B75] Li M. Gong J. Gao L. Zou T. Kang J. Xu H. (2022c). Advanced human developmental toxicity and teratogenicity assessment using human organoid models. Ecotoxicol. Environ. Saf. 235, 113429. 10.1016/j.ecoenv.2022.113429 35325609

[B76] Li M. Hou Z. Meng R. Hao S. Wang B. (2022a) Unraveling the potential human health risks from used disposable face mask-derived micro/nanoplastics during the COVID-19 pandemic scenario: A critical review. Environ. Int. 170, 107644. 10.1016/j.envint.2022.107644 36413926 PMC9671534

[B77] Li M. Zeng Y. Ge L. Gong J. Weng C. Yang C. (2022b). Evaluation of the influences of low dose polybrominated diphenyl ethers exposure on human early retinal development. Environ. Int. 163, 107187. 10.1016/j.envint.2022.107187 35313214

[B78] Liu Z. Cai M. Wu D. Yu P. Jiao Y. Jiang Q. (2020). Effects of nanoplastics at predicted environmental concentration on *Daphnia pulex* after exposure through multiple generations. Environ. Pollut. 256, 113506. 10.1016/j.envpol.2019.113506 31706756

[B79] Lu L. Wan Z. Luo T. Fu Z. Jin Y. (2018). Polystyrene microplastics induce gut microbiota dysbiosis and hepatic lipid metabolism disorder in mice. Sci. Total Environ. 631-632, 449–458. 10.1016/j.scitotenv.2018.03.051 29529433

[B80] Luo T. Wang C. Pan Z. Jin C. Fu Z. Jin Y. (2019a). Maternal polystyrene microplastic exposure during gestation and lactation altered metabolic homeostasis in the dams and their F1 and F2 offspring. Environ. Sci. Technol. 53 (18), 10978–10992. 10.1021/acs.est.9b03191 31448906

[B81] Luo T. Zhang Y. Wang C. Wang X. Zhou J. Shen M. (2019b). Maternal exposure to different sizes of polystyrene microplastics during gestation causes metabolic disorders in their offspring. Environ. Pollut. 255, 113122. 10.1016/j.envpol.2019.113122 31520900

[B82] Luo Y. Naidu R. Zhang X. Fang C. (2022). Microplastics and nanoplastics released from a PPE mask under a simulated bushfire condition. J. Hazard. Mater. 439, 129621. 10.1016/j.jhazmat.2022.129621 35878497

[B83] Mahler G. J. Esch M. B. Tako E. Southard T. L. Archer S. D. Glahn R. P. (2012). Oral exposure to polystyrene nanoparticles affects iron absorption. Nat. Nanotechnol. 7 (4), 264–271. 10.1038/nnano.2012.3 22327877

[B84] Mason S. A. Welch V. G. Neratko J. (2018). Synthetic polymer contamination in bottled water. Front. Chem. 6, 407. 10.3389/fchem.2018.00407 30255015 PMC6141690

[B85] Mattsson K. Johnson E. V. Malmendal A. Linse S. Hansson L-A. Cedervall T. (2017). Brain damage and behavioural disorders in fish induced by plastic nanoparticles delivered through the food chain. Sci. Rep. 7 (1), 11452. 10.1038/s41598-017-10813-0 28904346 PMC5597631

[B86] McCarthy J. Gong X. Nahirney D. Duszyk M. Radomski M. (2011). Polystyrene nanoparticles activate ion transport in human airway epithelial cells. Int. J. Nanomed 6, 1343–1356. 10.2147/IJN.S21145 PMC3133525 21760729

[B87] Nich C. Goodman S. B. (2014). Role of macrophages in the biological reaction to wear debris from joint replacements. J. Long. Term. Eff. Med. Implants 24 (4), 259–265. 10.1615/jlongtermeffmedimplants.2014010562 25747029 PMC4366682

[B88] Oberoi G. Garg A. (2021). Single-use plastics: A roadmap for sustainability? Supremo Amic. 24, 585.

[B89] OECD (2017). Test No. 318: Dispersion stability of nanomaterials in simulated environmental media, OECD guidelines for the testing of chemicals, section 3. Paris, France: OECD Publishing Paris.

[B90] Olivier V. Rivière C. Hindié M. Duval J. L. Bomila-Koradjim G. Nagel M. D. (2004). Uptake of polystyrene beads bearing functional groups by macrophages and fibroblasts. Colloids Surfaces B Biointerfaces 33 (1), 23–31. 10.1016/j.colsurfb.2003.08.008

[B91] Pitt J. A. Kozal J. S. Jayasundara N. Massarsky A. Trevisan R. Geitner N. (2018). Uptake, tissue distribution, and toxicity of polystyrene nanoparticles in developing zebrafish (*Danio rerio*). Aquat. Toxicol. 194, 185–194. 10.1016/j.aquatox.2017.11.017 29197232 PMC6959514

[B92] Prietl B. Meindl C. Roblegg E. Pieber T. R. Lanzer G. Fröhlich E. (2014). Nano-sized and micro-sized polystyrene particles affect phagocyte function. Cell. Biol. Toxicol. 30 (1), 1–16. 10.1007/s10565-013-9265-y 24292270 PMC4434214

[B93] Prüst M. Meijer J. Westerink R. H. S. (2020). The plastic brain: Neurotoxicity of micro- and nanoplastics. Part Fibre Toxicol. 17 (1), 24. 10.1186/s12989-020-00358-y 32513186 PMC7282048

[B94] Qu M. Liu Y. Xu K. Wang D. (2019). Activation of p38 MAPK signaling-mediated endoplasmic reticulum unfolded protein response by nanopolystyrene particles Adv. Biosyst. 3 (4), 1800325. 10.1002/adbi.201800325 32627431

[B95] Ragusa A. Svelato A. Santacroce C. Catalano P. Notarstefano V. Carnevali O. (2021). Plasticenta: First evidence of microplastics in human placenta. Environ. Int. 146, 106274. 10.1016/j.envint.2020.106274 33395930

[B96] Rahman A. Sarkar A. Yadav O. P. Achari G. Slobodnik J. (2021). Potential human health risks due to environmental exposure to nano- and microplastics and knowledge gaps: A scoping review. Sci. Total Environ. 757, 143872. 10.1016/j.scitotenv.2020.143872 33310568

[B97] Reddy A. S. Nair A. T. (2022). The fate of microplastics in wastewater treatment plants: An overview of source and remediation technologies. Environ. Technol. Innovation 28, 102815. 10.1016/j.eti.2022.102815

[B98] Revel M. Châtel A. Mouneyrac C. (2018). Micro(nano)plastics: A threat to human health? Curr. Opin. Environ. Sci. Health 1, 17–23. 10.1016/j.coesh.2017.10.003

[B99] Rodrigues M. O. Abrantes N. Gonçalves F. J. M. Nogueira H. Marques J. C. Gonçalves A. M. M. (2019). Impacts of plastic products used in daily life on the environment and human health: What is known? Environ. Toxicol. Pharmacol. 72, 103239. 10.1016/j.etap.2019.103239 31472322

[B100] Rossi G. Barnoud J. Monticelli L. (2014). Polystyrene nanoparticles perturb lipid membranes. J. Phys. Chem. Lett. 5 (1), 241–246. 10.1021/jz402234c 26276207

[B101] Saftig P. Haas A. (2016). Turn up the lysosome. Nat. Cell. Biol. 18 (10), 1025–1027. 10.1038/ncb3409 27684505

[B102] Salatin S. Maleki Dizaj S. Yari Khosroushahi A. (2015). Effect of the surface modification, size, and shape on cellular uptake of nanoparticles. Cell. Biol. Int. 39 (8), 881–890. 10.1002/cbin.10459 25790433

[B103] Sana S. S. Dogiparthi L. K. Gangadhar L. Chakravorty A. Abhishek N. (2020). Effects of microplastics and nanoplastics on marine environment and human health. Environ. Sci. Pollut. Res. 27 (36), 44743–44756. 10.1007/s11356-020-10573-x 32876819

[B104] Schwabl P. Köppel S. Königshofer P. Bucsics T. Trauner M. Reiberger T. (2019). Detection of various microplastics in human stool: A prospective case series. Ann. Intern Med. 171 (7), 453–457. 10.7326/M19-0618 31476765

[B105] Selvam S. Manisha A. Venkatramanan S. Chung S. Y. Paramasivam C. R. Singaraja C. (2020). Microplastic presence in commercial marine sea salts: A baseline study along tuticorin coastal salt pan stations, gulf of mannar, south India. Mar. Pollut. Bull. 150, 110675. 10.1016/j.marpolbul.2019.110675 31669711

[B106] Sepe R. Bollino F. Boccarusso L. Caputo F. (2018). Influence of chemical treatments on mechanical properties of hemp fiber reinforced composites. Compos B Eng. 133, 210–217. 10.1016/j.compositesb.2017.09.030

[B107] Shams M. Alam I. Mahbub M. S. (2021). Plastic pollution during COVID-19: Plastic waste directives and its long-term impact on the environment. Environ. Adv. 5, 100119. 10.1016/j.envadv.2021.100119 34604829 PMC8464355

[B108] Shim W. J. Hong S. H. Eo S. (2018). “Chapter 1 - marine microplastics: Abundance, distribution, and composition,” in Microplastic contamination in aquatic environments. Editor Zeng E. Y. (Elsevier), 1–26.

[B109] Sillanpää M. Sainio P. (2017). Release of polyester and cotton fibers from textiles in machine washings. Environ. Sci. Pollut. Res. 24 (23), 19313–19321. 10.1007/s11356-017-9621-1 28669092

[B110] Song W. Popp L. Yang J. Kumar A. Gangoli V. S. Segatori L. (2015) The autophagic response to polystyrene nanoparticles is mediated by transcription factor EB and depends on surface charge. J. Nanobiotechnology 13 (1), 87. 10.1186/s12951-015-0149-6 26596266 PMC4657241

[B111] Stock V. Böhmert L. Lisicki E. Block R. Cara-Carmona J. Pack L. K. (2019). Uptake and effects of orally ingested polystyrene microplastic particles *in vitro* and *in vivo* . Archives Toxicol. 93 (7), 1817–1833. 10.1007/s00204-019-02478-7 31139862

[B112] Tomazic‐Jezic V. J. Merritt K. Umbreit T. H. (2001). Significance of the type and the size of biomaterial particles on phagocytosis and tissue distribution J. Biomed. Mater. Res. Official J. Soc. Biomaterials, Jpn. Soc. Biomaterials, Aust. Soc. Biomaterials Korean Soc. Biomaterials 55 (4), 523–529. 10.1002/1097-4636(20010615)55:4<523::aid-jbm1045>3.0.co;2-g 11288080

[B113] Trainic M. Flores J. M. Pinkas I. Pedrotti M. L. Lombard F. Bourdin G. (2020). Airborne microplastic particles detected in the remote marine atmosphere. Commun. Earth Environ. 1 (1), 64. 10.1038/s43247-020-00061-y

[B114] Wang F. Salvati A. Boya P. (2018). Lysosome-dependent cell death and deregulated autophagy induced by amine-modified polystyrene nanoparticles. Open Biol. 8 (4), 170271. 10.1098/rsob.170271 29643148 PMC5936715

[B115] Wang F. Yu L. Monopoli M. P. Sandin P. Mahon E. Salvati A. (2013). The biomolecular corona is retained during nanoparticle uptake and protects the cells from the damage induced by cationic nanoparticles until degraded in the lysosomes. Nanomed Nanotechnol. Biol. Med. 9 (8), 1159–1168. 10.1016/j.nano.2013.04.010 23660460

[B116] Xia L. Alqahtani S. Ferreira C. R. Aryal U. K. Biggs K. Shannahan J. H. (2022). Modulation of pulmonary toxicity in metabolic syndrome due to variations in iron oxide nanoparticle-biocorona composition. Nanomater. (Basel) 12 (12), 2022. 10.3390/nano12122022 PMC9230893 35745361

[B117] Xia W. Rao Q. Deng X. Chen J. Xie P. (2020). Rainfall is a significant environmental factor of microplastic pollution in inland waters. Sci. Total Environ. 732, 139065. 10.1016/j.scitotenv.2020.139065 32422477

[B118] Xie Y. Li Y. Feng Y. Cheng W. Wang Y. (2022). Inhalable microplastics prevails in air: Exploring the size detection limit. Environ. Int. 162, 107151. 10.1016/j.envint.2022.107151 35228011

[B119] Yang Y. Shao H. Wu Q. Wang D. (2020). Lipid metabolic response to polystyrene particles in nematode *Caenorhabditis elegans* . Environ. Pollut. 256, 113439. 10.1016/j.envpol.2019.113439 31672355

[B120] Yao Y. Glamoclija M. Murphy A. Gao Y. (2022). Characterization of microplastics in indoor and ambient air in northern New Jersey. Environ. Res. 207, 112142. 10.1016/j.envres.2021.112142 34597660

[B121] Yee M. S. Hii L. W. Looi C. K. Lim W. M. Wong S. F. Kok Y. Y. (2021). Impact of microplastics and nanoplastics on human health. Nanomater. (Basel) 11 (2), 496. 10.3390/nano11020496 PMC7920297 33669327

[B122] Yong C. Q. Valiyaveettil S. Tang B. L. (2020). Toxicity of microplastics and nanoplastics in mammalian systems. Int. J. Environ. Res. Public Health 17. 10.3390/ijerph17051509 PMC7084551 32111046

[B123] Zhang R. Silic M. R. Schaber A. Wasel O. Freeman J. L. Sepúlveda M. S. (2020). Exposure route affects the distribution and toxicity of polystyrene nanoplastics in zebrafish. Sci. Total Environ. 724, 138065. 10.1016/j.scitotenv.2020.138065 32272399

[B124] Zhang S. Wang J. Liu X. Qu F. Wang X. Wang X. (2019). Microplastics in the environment: A review of analytical methods, distribution, and biological effects. TrAC Trends Anal. Chem. 111, 62–72. 10.1016/j.trac.2018.12.002

[B125] Zhang Z. Gao S-H. Luo G. Kang Y. Zhang L. Pan Y. (2022). The contamination of microplastics in China's aquatic environment: Occurrence, detection and implications for ecological risk. Environ. Pollut. 296, 118737. 10.1016/j.envpol.2021.118737 34954308

